# Mechanism of Activation of Mechanistic Target of Rapamycin Complex 1 by Methionine

**DOI:** 10.3389/fcell.2020.00715

**Published:** 2020-08-11

**Authors:** Munehiro Kitada, Jing Xu, Yoshio Ogura, Itaru Monno, Daisuke Koya

**Affiliations:** ^1^Department of Diabetology and Endocrinology, Kanazawa Medical University, Uchinada, Japan; ^2^Division of Anticipatory Molecular Food Science and Technology, Medical Research Institute, Kanazawa Medical University, Uchinada, Japan

**Keywords:** methionine, *S*-adenosyl methionine, mechanistic target of rapamycin complex 1, autophagy, SAMTOR, phosphatase 2A methylation

## Abstract

Nutrients are closely involved in the regulation of lifespan and metabolic health. Cellular activities, such as the regulation of metabolism, growth, and aging, are mediated by a network of nutrients and nutrient-sensing pathways. Among the nutrient-sensing pathways, the mechanistic target of rapamycin complex 1 (mTORC1) acts as the central regulator of cellular functions, which include autophagy. Autophagy plays a significant role in the removal of protein aggregates and damaged or excess organelles, including mitochondria, to maintain intracellular homeostasis, which is involved in lifespan extension and cardiometabolic health. Moreover, dietary methionine restriction may have a beneficial effect on lifespan extension and metabolic health. In contrast, methionine may activate mTORC1 and suppress autophagy. As the mechanism of methionine sensing on mTORC1, SAMTOR was identified as a sensor of *S*-adenosyl methionine (SAM), a metabolite of methionine, in the cytoplasm. Conversely, methionine may activate the mTORC1 signaling pathway through the activation of phosphatase 2A (PP2A) because of increased methylation in response to intracellular SAM levels. In this review, we summarized the recent findings regarding the mechanism via which methionine activates mTORC1.

## Introduction

All organisms adapt and respond to the nutrients available in the environment. Cellular activities, including the regulation of metabolism, cell growth, and aging, are mediated by a network that comprised nutrients and nutrient-sensing pathways (Efeyan et al., 2015). Dietary interventions, such as calorie or dietary restriction and protein restriction, have been widely explored for their impact on lifespan extension or the prevention of age-related diseases through effects on cardiometabolic health. Calorie or dietary restriction without malnutrition has been demonstrated to extend the lifespan of organisms and improve their cardiometabolic health (Colman et al., 2009; Fontana et al., 2010; Mattison et al., 2012, 2017). However, recent studies have reported that protein restriction, rather than calorie or dietary restriction, is more strongly involved in the lifespan extension and cardiometabolic health (Nakagawa et al., 2012; Levine et al., 2014; Solon-Biet et al., 2014; Simpson et al., 2017; Kitada et al., 2019). Moreover, accumulated evidence from experimental studies indicates that the restriction of specific amino acids, such as branched-chain amino acids (BCAAs) or methionine, promotes longevity and cardiometabolic health (Fontana et al., 2016; Lee et al., 2016; Cummings et al., 2018; Kitada et al., 2019), which possibly mediates the benefits of protein restriction.

Among the nutrient-sensing pathways, the mechanistic target of rapamycin complex 1 (mTORC1) is a serine/threonine protein kinase that acts as the central regulator of cell growth and metabolism in response to the changes in nutrients or growth factors (Kim and Guan, 2019). Numerous studies on the pharmacological inhibition of mTORC1 by rapamycin have demonstrated the lifespan-extension benefit of this approach (Harrison et al., 2009; Anisimov et al., 2011; Wilkinson et al., 2012; Miller et al., 2014; Zhang et al., 2014), which suggest that mTORC1 is closely involved in lifespan regulation. The mechanism via which the suppression of mTORC1 leads to lifespan extension includes the induction of the autophagy (Kim and Guan, 2019). Autophagy is a lysosomal degradation pathway that plays an important role in the removal of protein aggregates and damaged or excess organelles, such as mitochondria, to maintain homeostasis and cell function (Mizushima et al., 2008). An appropriate autophagy may protect cells against various age-related stress conditions, which results in lifespan extension and cardiometabolic health (Wong et al., 2020). mTORC1 has been recognized as a crucial regulator of autophagy, and amino acids are one of the strong factors that affect mTORC1 activation (Kim and Guan, 2019). Thus, the beneficial effect of protein restriction on lifespan extension may be mediated through the induction of autophagy via the suppression of mTORC1 under amino-acid restriction. Recent findings have clarified that essential amino acids, including BCAAs or methionine, are possibly related to the regulation of the aging process, lifespan, and cardiometabolic health through multiple physiological and molecular mechanisms. In particular, the mechanisms underlying the role of methionine in the regulation of aging or lifespan have been widely investigated through dietary intervention via the application of a methionine restriction diet. Among these mechanisms, the involvement of methionine in the regulation of mTORC1 and autophagy has been elucidated based on the results of those studies. In the current review, we summarized the recent findings regarding the mechanism of mTORC1 activation by methionine.

## Role of Rags on the Regulation of mTORC1 Activity by Amino-Acid Sensing

The mTORC1 activity is regulated by several molecules in response to changes in nutrients, including amino acids and growth factors. Moreover, the upstream component of the amino-acid–sensing pathway of mTORC1 is complicated (Kim and Guan, 2019). The regulation of mTORC1 activity by amino acids occurs through the translocation and localization of mTORC1 to lysosomes. The heterodimers of low-molecular-weight GTPases, RagA or B, and RagC, or D (Kim et al., 2008; Sancak et al., 2008; Anandapadamanaban et al., 2019), which are localized in lysosomes, play an important role in the activation of mTORC1 by amino acids. RagA and RagC exist as a dimer, and the GTP-bound form of RagA is its active form, whereas the GDP-bound form of RagC is its active form. In the presence of amino acids, these proteins function as activated GTP-RagA or GDP-RagC. In contrast, under amino-acid starvation, they function as a combination of inactivated GDP-RagA or GTP-RagC. The activated Rag dimer binds to Raptor, which is a major component of mTORC1, and participates in the translocation and localization of mTORC1 from the cytoplasm to lysosomes. Thereafter, in the lysosome, mTORC1 is activated by GTP-Rheb.

**FIGURE 1:**
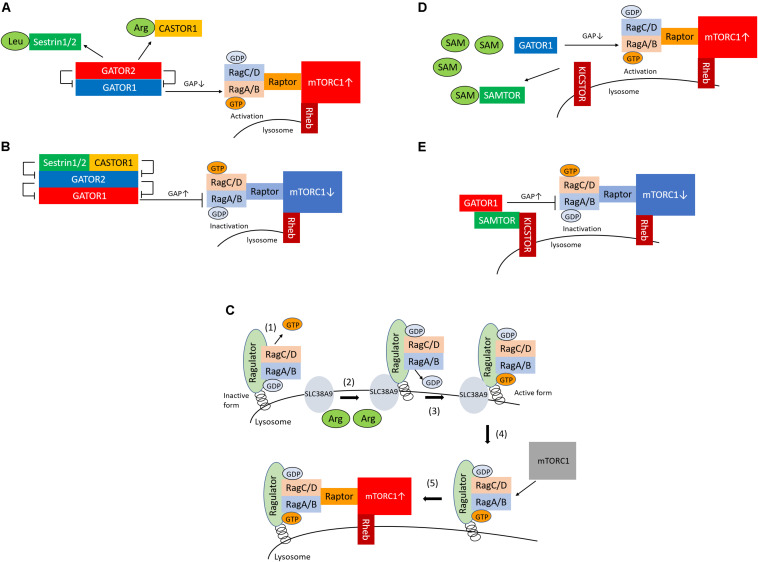
Sestrin1/2, CASTOR1, and SAMTOR are cytosolic sensors of leucine, arginine, and SAM for the regulation of mTORC1. **(A)** Sestrin1/2 and CASTOR1 sense leucine and arginine in the cytoplasm, respectively. Upon binding of leucine or arginine to sestrin1/2 and CASTOR1, these proteins dissociate from GATOR2, which releases their suppressive effect on GATOR2. GATOR2 suppresses GATOR1, which consequently activates mTORC1 via GTP-bounded RagA formation by GAP inactivation. **(B)** In conditions of low leucine and arginine levels, sestrin1/2 and CASTOR1 suppress GATOR2 (the negative regulator of GATOR1), which leads to the activation of GATOR1 and results in the suppression of mTORC1 via GAP activation of RagA. **(C)** (1) Ragulator releases GTP from RagC; (2) SLC38A9 is activated by arginine in the lysosome; (3) SLC38A9 converts RagA from the GDP- to the GTP-bound state, leading to the activation of the Rags; (4) Ragulator and SLC38A9 recruit the mTORC1 to the lysosomal surface; and (5) mTORC1 is activated. **(D)** SAMTOR senses SAM in the cytoplasm. Upon the binding of SAM to SAMTOR, SAMTOR dissociates from GATOR1. The disruption of the SAMTOR–GATOR1 complex leads to the inactivation of GATOR1, which results in mTORC1 activation via the inhibition of GAP activation and increased GTP binding to RagA. **(E)** In conditions of low levels of SAM, the SAMTOR–GATOR1 complex suppresses mTORC1 activity. SAM, *S*-adenosyl methionine; mTORC1, mechanistic target of rapamycin complex 1; GAP, GTPase-activating protein.

The GATOR1 and GATOR2 complexes are recognized as Rag regulators and are localized in the cytoplasm (Bar-Peled et al., 2013). GATOR1 is a complex composed of three proteins, DEPDC5, NPRL2, and NPRL3, and has RagA-binding ability and GTPase-activating protein (GAP) activity for RagA (Shen et al., 2018). DEPDC5 of GATOR1 contains a GAP domain, which binds directly to RagA, thus inactivating it. However, deletion of the GATOR1 component results in the amino-acid–independent localization and activation of mTORC1 in the lysosome, which demonstrates that GATOR1 is a negative regulator of mTORC1. In contrast, GATOR2 is a complex consisting of five proteins, Sec13, Seh1L, WDR24, WDR59, and Mios (Bar-Peled et al., 2013). GATOR2 binds to GATOR1; GATOR2 acts as the positive regulator of mTORC1 by suppressing the GAP activity of GATOR1. Leucine and arginine bind to sestrin1/2 and CASTOR1, respectively, and sestrin1/2 and CASTOR1 are also recognized as sensors of leucine or arginine (Figure 1A). Amino-acid–bound sensor proteins dissociate from GATOR2, thus losing their ability to inactivate GATOR2 (Figure 1A; Chantranupong et al., 2014, 2016; Parmigiani et al., 2014; Saxton et al., 2016a, b; Wolfson et al., 2016). Consequently, the activated GATOR2 triggers the activation of mTORC1 through the inactivation of GATOR1. Conversely, during leucine or arginine starvation, sestrin1/2 and CASTOR1 bind to GATOR2 and inactivate GATOR2, which results in mTORC1 inactivation via an increase in the RagA GAP activity of GATOR1 (Figure 1B).

In addition to the cytosolic amino-acid–sensing branch, Shen and Sabatini reported that Ragulator and SLC38A9 are two critical regulators of the activation of mTORC1 as the lysosomal amino-acid–sensing branch (Shen and Sabatini, 2018) (Figure 1C). Ragulator tethers the Rag heterodimer to the lysosomal surface, and the SLC38A9 transmembrane protein is a lysosomal arginine sensor that stimulates mTORC1 activity through the regulation of Rags. Ragulator and SLC38A9 are guanine exchange factors that lead the Rags toward the active form (Shen and Sabatini, 2018). Ragulator triggers GTP release from RagC, thus lifting the locked inactivated state of the Rags (Shen and Sabatini, 2018). Upon arginine binding, SLC38A9 converts RagA from the GDP- to the GTP-bound state, leading to the activation of the Rags (Shen and Sabatini, 2018). Thus, Ragulator and SLC38A9 activate mTORC1 by recruiting it to the lysosomal surface via Rag activation in response to arginine levels in the lysosome. Moreover, v-ATPase interacts with Ragulator, Rags, and SLC38A9 and is involved both in amino-acid–sensing and in efflux from the lysosome (Zoncu et al., 2011; Abu-Remaileh et al., 2017; Wyant et al., 2017). However, it remains unknown whether v-ATPase senses amino acids.

## Methionine-Induced mTORC1 Activation and the Role of Samtor as a Sam Sensor That Provides a Link to the Methionine Metabolism

The KICSTOR complex is one of the regulators of mTORC1 and comprises kaptin (KPTN), the integrin-α FG-GAP repeat-containing protein 2 (ITFG2), C12orf66, and seizure threshold 2 (SZT2) (Wolfson et al., 2017). C7orf60 was identified as an interacting protein of GATOR1 and was subsequently renamed SAMTOR (Gu et al., 2017). The overexpression of SAMTOR suppresses mTORC1 activity, which indicates that SAMTOR is a negative regulator of mTORC1. SAM is converted from methionine, and methionine starvation reduces the concentration of SAM in the cytoplasm. When present of SAM, SAM binds to SAMTOR, which then dissociates from GATOR1 (Figure 1D). The disruption of the SAMTOR–GATOR1 complex leads to the inactivation of GATOR1, which then results in mTORC1 activation through the inhibition of GAP activation and increased binding of GTP to RagA. In contrast, methionine starvation reduces SAM levels below the dissociation constant of the SAM–SAMTOR complex, thus promoting SAMTOR–GATOR1 binding and, in turn, suppressing mTORC1 activity (Figure 1E). However, loss of SAMTOR activates mTORC1, even in conditions of methionine starvation. In addition, SAMTOR mutants that cannot bind to SAM fail to transmit methionine sufficiently to mTORC1, therefore suppressing mTORC1. These results indicate that SAMTOR serves as a SAM sensor in the methionine-mediated mTORC1 activation.

## Role of the Induction of the Methylation of PP2A by Sam in mTORC1 Activation

**FIGURE 2:**
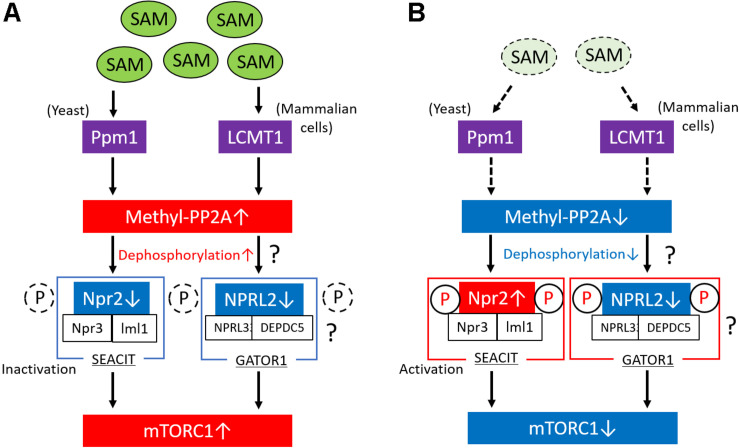
Regulation of mTORC1 via the methylation of PP2A in response to SAM. **(A)** In conditions of high levels of intracellular SAM in yeast, Ppm1 induces the methylation of the catalytic subunit of PP2A in response to SAM concentration. The activated (methylated) form of PP2A suppresses Npr2 through its dephosphorylation. The complex consisting of Npr2, Npr3, and Iml1 (SEACIT) is a negative regulator of mTORC1; therefore, the suppression of SEACIT via the dephosphorylation of Npr2 results in the activation of mTORC1. In mammalian cells, LCMT1 induces the methylation of the catalytic subunit of PP2A in response to SAM concentration, leading to the activation of mTORC1, possibly through the activation of GATOR1. Moreover, in mammalian cells, PP2A possibly regulates the phosphorylation levels of NPRL2 in response to SAM levels. **(B)** Lower SAM levels reduce the methylation levels of PP2A in yeast and mammalian cells and promote the activation of Npr2 via its phosphorylation, which results in the suppression of mTORC1 activity. In mammalian cells, PP2A possibly regulates the phosphorylation levels of NPRL2 in response to SAM levels. mTORC1, mechanistic target of rapamycin complex 1; PP2A, phosphatase 2A; SAM, *S*-adenosyl methionine; GAP, GTPase-activating protein; LCMT1, leucine carboxyl methyltransferase 1.

The study performed by Sutter et al. also showed that methionine regulates the mTORC1 signaling pathway and autophagy through the regulation of the methylation status of phosphatase 2A (PP2A) in yeast (Sutter et al., 2013; Laxman et al., 2014). In the presence of high levels of intracellular SAM, Ppm1 induces the methylation of the catalytic subunit of PP2A in response to SAM concentration. PP2A is activated by its methylation; thereafter, methylated PP2A can suppress Npr2 through its dephosphorylation, which results in mTORC1 activation and the suppression of autophagy (Figure 2A). The complex consisting of Npr2, Npr3, and Iml1 (NPRL2, NPRL3, and DEPDC5 in mammals, respectively) is termed SEACIT in yeast (GATOR1 in mammals, as described above) (Panchaud et al., 2013) and functions as a negative regulator of mTORC1 via a GAP activity toward the yeast Rag orthologs, that is, Gtr1/2 (Rags family in mammals) (Gao and Kaiser, 2006). Therefore, suppression of SEACIT by the dephosphorylation of Npr2 induced by the activation of PP2A results in the activation of mTORC1. In contrast, lower SAM levels in cells reduce the methylation levels of PP2A and promote the phosphorylation of Npr2, which results in the suppression of mTORC1 activity and the induction of autophagy (Figure 2B). In mammalian cells, the methylation of PP2A is catalyzed by a specific *S-*adenosyl methionine (SAM)–dependent methyltransferase, the leucine carboxyl methyltransferase 1 (LCMT1) (Stanevich et al., 2011). Activated PP2A possibly dephosphorylates NPRL2 and results in mTORC1 activation in mammalian cells; however, no report has shown whether PP2A is directly involved in the regulation of the phosphorylation state of NPRL2. Therefore, further studies are necessary to clarify this issue.

We also reported that a low-protein diet ameliorates diabetes-induced kidney injury and that dietary methionine abrogates the beneficial effects of a low-protein diet in diabetic kidneys (Kitada et al., 2020). More specifically, diabetic rats that were fed a low-protein + methionine diet exhibited increased expression of LCMT1 and methyl-PP2A compared with control (standard-diet–fed) and low-protein-diet–fed diabetic rats, which was accompanied by an increase in renal SAM levels. Although the expression of glycine *N*-methyltransferase (Gnmt), which is a SAM-converted enzyme, was decreased in diabetic rat kidneys, changes in renal SAM levels were dependent on the dietary methionine content (Kitada et al., 2020). Consistent with the alteration of LCMT1 and methyl-PP2A, mTORC1 activation and autophagy suppression were observed in standard-diet–fed and low-protein + methionine–fed diabetic rats. Furthermore, we also used cultured human kidney-2 cells to confirm that the administration of SAM-induced methylated PP2A increased the expression of methyl-PP2A and activated mTORC1 (Kitada et al., 2020). However, the involvement of SAM-induced methylated PP2A in mTORC1 activation through NPRL2 and the activation of the negative regulator of mTORC1 by its increased phosphorylation, such as that observed for Npr2 in yeast, remain unknown.

## Methionine Activates mTORC1 Through TAS1R1/TAS1R3

Nelson et al. previously identified a mammalian amino-acid taste receptor, the taste 1 receptor member 1 (TAS1R1)/taste 1 receptor member 3 (TAS1R3) heterodimer, which is a cell-surface G-protein–coupled receptor (Nelson et al., 2002). This receptor broadly functions as an amino-acid sensor that responds to most of the 20 standard amino acids. Upon sensing amino acids, this receptor activates mTORC1 through the activation of phospholipase C, the increase in intracellular calcium, and the activation of the mitogen-activated protein kinase 1/mitogen-activated protein kinase 3 (Wauson et al., 2015). TAS1R1–TAS1R3 is required for the amino-acid–induced mTORC1 localization to the lysosome, which is a necessary step in mTORC1 activation. Several reports have demonstrated that TAS1R1–TAS1R3 may serve as a sensor of extracellular methionine and that it activates mTORC1 in cultured C2C12 cells and bovine epithelial cells (Zhou et al., 2016, 2018).

## Discussion

In this study, we described the recent findings regarding the mechanism via which methionine induces the activation of mTORC1. mTORC1 may be activated by sensing SAM rather than methionine. A previous report by Obata et al. provided evidence that SAM, rather than methionine, may be the main contributor to the aging process (Obata and Miura, 2015). Those authors showed that increasing SAM catabolism via the action of glycine *N*-methyltransferase (Gnmt) extends the lifespan in *Drosophila*. In particular, SAM is upregulated in older flies, even if the transcription of Gnmt is induced in a forkhead box O (FOXO)–dependent manner. However, overexpression of Gnmt suppresses the age-dependent increase in SAM and extends lifespan in *Drosophila*. In addition, metabolic impairment, such as insulin resistance in obesity, is closely involved in the aging process. A previous report demonstrated that plasma SAM concentrations were related to higher fasting insulin levels, the homeostasis model assessment of insulin resistance, and the tumor necrosis factor α in a cross-sectional study that involved subjects with metabolic syndrome (Lind et al., 2018). Another report also revealed that plasma SAM, and not methionine, is independently related to fat mass and truncal adiposity in a cross-sectional study involving elderly individuals (Elshorbagy et al., 2013); in contrast, overfeeding increases serum SAM in proportion to the fat mass gained (Elshorbagy et al., 2016). Thus, the upregulation of SAM associated with overfeeding or metabolic dysfunction may be involved in whole-body metabolic impairment. These data indicate that increased levels of SAM in the process of methionine metabolism may be related to the stimulation of aging and metabolic impairment, including insulin resistance, which is particularly associated with obesity. Previous reports have shown that dietary methionine restriction extends the lifespan or improves cardiometabolic health (Orentreich et al., 1993; Miller et al., 2005; Hasek et al., 2010; Plaisance et al., 2011; Johnson and Johnson, 2014; Lee et al., 2014; Stone et al., 2014). The effect of methionine restriction on lifespan extension or cardiometabolic health may be exerted through multiple mechanisms, including antioxidative stress, the production of hydroxy sulfates, the downregulation of GH/insulin growth factor 1 signaling, the production of fibroblast growth factor 21, the suppression of mTORC1, and the induction of autophagy (Kitada et al., 2019). Among them, the suppression of mTORC1 is induced by decreasing SAM levels. Therefore, the regulation of SAM levels and sensing of SAM in the cytoplasm may be key factors in the mechanism of lifespan extension, which may be mediated by the regulation of mTORC1. Because the selective suppression of mTORC1 induced by SAM may be a therapeutic target for aging, metabolic impairment, or aging-related disease, further studies are necessary to address these issues.

## Author Contributions

MK designed the manuscript, the guarantor of this work, and wrote and edited the manuscript. JX, YO, IM, and DK contributed to the discussion. All authors contributed to the article and approved the submitted version.

## Conflict of Interest

Boehringer Ingelheim, Mitsubishi Tanabe Pharma, Kyowa Kirin, Taisho Pharmaceutical Co., Ltd., and Ono Pharmaceutical Co., Ltd. contributed to establishing the Division of Anticipatory Molecular Food Science and Technology. The authors declare that the research was conducted in the absence of any commercial or financial relationships that could be construed as a potential conflict of interest.
